# Emerging Roles and Mechanisms of lncRNA FOXD3-AS1 in Human Diseases

**DOI:** 10.3389/fonc.2022.848296

**Published:** 2022-02-25

**Authors:** Qinfan Yao, Xiuyuan Zhang, Dajin Chen

**Affiliations:** ^1^ Kidney Disease Center, The First Affiliated Hospital, College of Medicine, Zhejiang University, Hangzhou, China; ^2^ Key Laboratory of Kidney Disease Prevention and Control Technology, Hangzhou, China; ^3^ National Key Clinical Department of Kidney Diseases, Institute of Nephrology, Zhejiang University, Hangzhou, China; ^4^ Zhejiang Clinical Research Center of Kidney and Urinary System Disease, Hangzhou, China

**Keywords:** long noncoding RNA, FOXD3-AS1, clinicopathological feature, function, mechanism

## Abstract

Numerous long noncoding RNAs (lncRNAs) have been identified as powerful regulators of human diseases. The lncRNA FOXD3-AS1 is a novel lncRNA that was recently shown to exert imperative roles in the initialization and progression of several diseases. Emerging studies have shown aberrant expression of FOXD3-AS1 and close correlation with pathophysiological traits of numerous diseases, particularly cancers. More importantly, FOXD3-AS1 was also found to ubiquitously impact a range of biological functions. This study aims to summarize the expression, associated clinicopathological features, major functions and molecular mechanisms of FOXD3-AS1 in human diseases and to explore its possible clinical applications.

## Introduction

Based on the in-depth advance of high-throughput sequencing technologies, an emerging number of long non-coding RNAs (lncRNAs) has been identified over recent decades (1–5). LncRNA is a novel type of non-coding RNA molecules with over 200 bp (6, 7), accounting for the largest proportion of non-coding RNAs (ncRNA) (8–10). Due to the continuous investigation of lncRNAs, it is believed that lncRNAs are closely related to the occurrence and development of tumors and other diseases (11–15). Several studies have corroborated that lncRNAs are extensively implicated in a range of cellular processes, such as chromatin and genome modifications, transcription activation and interference, nuclear transport (16–20), as well as cell growth, differentiation, and apoptosis. Moreover, lncRNA-based clinical applications have been increasingly explored over the last few years and several mechanisms for such applications have been identified (21–23).

LncRNA forkhead box D3 antisense 1 (FOXD3-AS1), an antisense transcript of the protein-coding gene FOXD3, is a recently discovered lncRNA located in chromosome 1p31.3. Growing evidence reports that FOXD3-AS1 is abnormally expressed in many disease types and its expression seems to be closely associated with significant clinical features. Functional assays demonstrated that FOXD3-AS1 is a crucial regulator in a wide range of biological functions in disease. Therefore, these properties rendered FOXD3-AS1 as a promising biomarker for various applications, including diagnosis, treatment, and prognosis of specific diseases. In this review, we aim to recapitulate the abnormal expression, clinical features, biological roles, corresponding mechanisms, and future clinical applications of FOXD3-AS1 in various human diseases.

## The Role Of lncRNA FOXD3-AS1 In Diseases

Increasing evidence has shown that lncRNA FOXD3-AS1 is abnormally expressed in various human diseases, including lung cancer (24–28), breast cancer (29, 30), cervical cancer (31, 32), nasopharyngeal carcinoma (33, 34), osteosarcoma (35), colorectal cancer (36), melanoma (37, 38), liver cancer (39), thyroid cancer (40), neuroblastoma (41), glioma (42), allergic rhinitis (43), retinal infection with Toxoplasma gondii-ocular toxoplasmosis (44), ischemic stroke (45), myocardial ischemia (46, 47) and acute respiratory distress syndrome (48). Further studies demonstrated that aberrant expression of FOXD3-AS1 is closely associated to clinicopathological characteristics, such as tumor size, tumor grade, distant lymph node metastasis, differentiation of tumor tissues, overall survival, progression-free survival, and survival time of patients (**Table 1**). Moreover, the specific role and related molecular mechanisms of FOXD3-AS1 in the occurrence and development of diseases are shown in **Table 1**.

**Table 1:** The expression, clinical characteristics, and mechanisms of FOXD3-AS1 in disease.

Disease type	Expression	Role	Clinical characteristics	Cell lines	Human samples	Functions	Related mechanisms	Refs
non-small cell lung cancer	upregulated	tumor promoter	/	A549, H1229, and SPC-A1	30 patients from Affiliated Nanhai Hospital, Southern Medical University, 40 patients from Hwamei Hospital, and 25 patients from Peking University Shenzhen Hospital	cell proliferation, apoptosis, invasion, and chemo-resistance	miR-135a-5p, miR-127-3p, MED28, ELAVL1, PI3K, Akt, and CDK6	34733371, 32742197, 32196603, 34605863
non-small cell lung cancer	downregulated	tumor suppressor	lymph node metastasis, and tumor grade	H1299, NCI-H460, A549, and L9981	50 patients from Shenzhen University General Hospital	cell proliferation, invasion, and EMT	miR-150, and SRCIN1	32924985
breast cancer	upregulated	tumor promoter	survival probability, tumor size, and distant metastasis	MDA-MB-231, BT549, T47D and MCF-7	19 patients from the First Affiliated Hospital of Wenzhou Medical University	cell proliferation, invasion, migration, and chemo-resistance	miR-363, TFF1, PI3K, and Akt	34424807, 31017311
cervical cancer	upregulated	tumor promoter	tumor differentiation, tumor size, lymph node metastasis, distant metastasis, overall survival rate, and International Federation of Gynecology and Obstetrics stage	HeLa, SiHa, C33A, SW756, ME-180, Caski, and HT-3	60 patients from the Hengshui People's Hospital, and 146 patients from The First Affiliated Hospital, Heilongjiang University of Chinese Medicine	cell proliferation, invasion, migration, and apoptosis	miR-128-3p, miR-296-5p, LIMK1, SP1, and HMGA1	33760158, 32959937
nasopharyngeal carcinoma	upregulated	tumor promoter	TNM stage, and pathological type	C666-1, and HK-1	52 patients from the Taihe Hospital, Hubei University of Medicine	cell proliferation, invasion, migration, apoptosis, and stemness	miR-135a-5p, microRNA-185-3p, and FOXD3	33204001, 33336076
osteosarcoma	upregulated	tumor promoter	/	U2OS, MG-63, HOS, SAOS2, and 143B	52 patients from the First Affiliated Hospital of Chongqing Medical University	cell migration, invasion, and EMT	miR-296-5p, ELF1, and ZCCHC3	33204608
colon adenocarcinoma	upregulated	tumor promoter	tumor differentiation, TNM stage, lymph node metastasis, poor prognosis, overall survival rate and progression-free survival rate	HCT116, and SW1116	78 patients from Tongren Hospital, Shanghai Jiao Tong University School of Medicine	cell proliferation, invasion, migration, and apoptosis	miR-135a-5p, and SIRT1	32932277, 31058315
melanoma	upregulated	tumor promoter	lymphatic metastasis, tumor size, AJCC stage, and overall survival	A2058, SK-MEL-28, SK-MEL-1, SK-MEL-2, and A375	47 patients from Weihai Central Hospital	cell proliferation, invasion, migration, and apoptosis	miR-127-3p, miR-325, FJX1, and MAP3K2	32354225, 31541886
hepatocellular carcinoma	upregulated	tumor promoter	poor prognosis	Huh7, Huh6, and SK-HEP-1	68 patients from Affiliated Hospital of Hebei University	cell proliferation, invasion, and migration	miR-335, RICTOR, and AKT	32191537
thyroid cancer	upregulated	tumor promoter	/	FTC-133, SW579, TPC-1, and 8505C	30 patients from Peking Union Medical College Hospital	cell proliferation, invasion, and migration	miR-296-5p, TGF-β1, and Smads	31678422
neuroblastoma	downregulated	tumor suppressor	tumor differentiation, International Neuroblastoma Staging System (INSS) stage, and MYCN amplification	NB-1643, SK-N-BE (2), NB-1691, IMR32, and BE (2)-C	/	cell proliferation, invasion, migration, differentiation, and chemo-sensitivity	CTCF, and PARP1	29398485
glioma	upregulated	tumor promoter	WHO grade, histologic grade, poor prognosis, and overall survival	U87, A172 and U251	44 patients from Changzheng Hospital (Shanghai, China)	cell proliferation, invasion, and migration	FOXD3	27829996
ischemia stroke	upregulated	/	/	N2a	/	cell apoptosis	miR-765, and BCL2L13	33068927
myocardial disease	upregulated	/	/	H9C2, and AC16	/	cell apoptosis	NF-κB, iNOS, and COX2	31632535 ,32973515
acute respiratory distress syndrome	upregulated	/	/	A549, and Beas2B	/	cell apoptosis	miR-150, and p53	28655711
allergic rhinitis	downregulated	/	/	NECs	25 patients from The Second Affiliated Hospital of Nanchang University	Th2 type immunoreaction	IL-25	32671514
retinal infection with Toxoplasma gondii-ocular toxoplasmosis	downregulated	/	/	human retinal Müller cells	/	/	/	31547203

In the following section, we recapitulated the role of FOXD3-AS1 in different disease types, including dysregulated expression, related clinicopathological features and biological functions.

### Cancer

#### Lung Cancer

Lung cancer is a major public health problem worldwide, while non-small cell lung cancer (NSCLC) represents about 80–90% of all cases (49–53). Indeed, NSCLC has a high mortality rate, and the 5-year survival rate of these patients remain challenging (54–57). Despite advancements in early detection and treatment of NSCLC, the identification of molecular markers associated with patient survival is still necessary (53, 58–60). Multiple studies have demonstrated that FOXD3-AS1 is overexpressed in NSCLC tissues and cell lines (H1299, A549 and SPC-A1 cells) (24, 26–28). In addition, FOXD3-AS1 exerts pro-cancer effects *in vitro* and *in vivo* by regulating cell proliferation, migration, apoptosis as well as chemo-resistance. Contradictorily, Ji T et al. first proposed that FOXD3-AS1 was down-regulated in NSCLC tissues and H1299, NCI-H460, A549 and L9981 cell lines, while FOXD3-AS1 levels were inversely correlated with aggressive lymph node metastasis and tumor grade (25). Later, FOXD3-AS1 was confirmed to play an onco-suppressive role by inhibiting cell proliferation and invasion of H1299 and A549 cells. It is worth noting that the contradictory effect of FoxD3-AS1 in this study was only assessed *in vitro* and may be partly due to the remarkably heterogeneous properties of lung cancers. Therefore, further verification of FoxD3-AS1 on animal models of lung cancer is still required (61–65).

#### Breast Cancer

Breast cancer has a high incidence and mortality in women (66, 67). Accumulating evidence has suggested that aberrant expression of lncRNAs is implicated in the tumorigenesis of breast cancer (68–71). FoxD3-AS1 expression was found to be significantly upregulated in breast cancer tissues, in addition to T47D, MCF7, BT549, and MDA-MB-231 cells. Interestingly, its overexpression correlated with survival probability, tumor size, and distant metastasis. Moreover, FoxD3-AS1 has been proposed to serve as a novel tumor promoter in the development and progression of breast cancer by enhancing cell proliferation, migration, invasion and tamoxifen (TMX) resistance (29, 30).

#### Cervical Cancer

Although vaccination against HPV and cervical cancer screening have remarkably reduced cervical cancer incidence and mortality (30, 72, 73), this cancer is still the most prevalent malignancy in women, with high mortality rate (74–77). Several studies have demonstrated that FOXD3-AS1 is markedly upregulated in cervical cancer tissues and cell lines such as HeLa, SiHa, Caski, SW756, C33A, ME-180, and HT-3 (31, 32). High levels of FOXD3-AS1 were linked to poorly differentiated tumors, large tumors, positive lymph node metastasis, distant metastasis, and worse International Federation of Gynecology and Obstetrics stage. FOXD3-AS1 displayed pro-oncogenic capacity by facilitating cell proliferation, apoptosis, migration and invasion.

#### Nasopharyngeal Carcinoma

**Figure 1:**
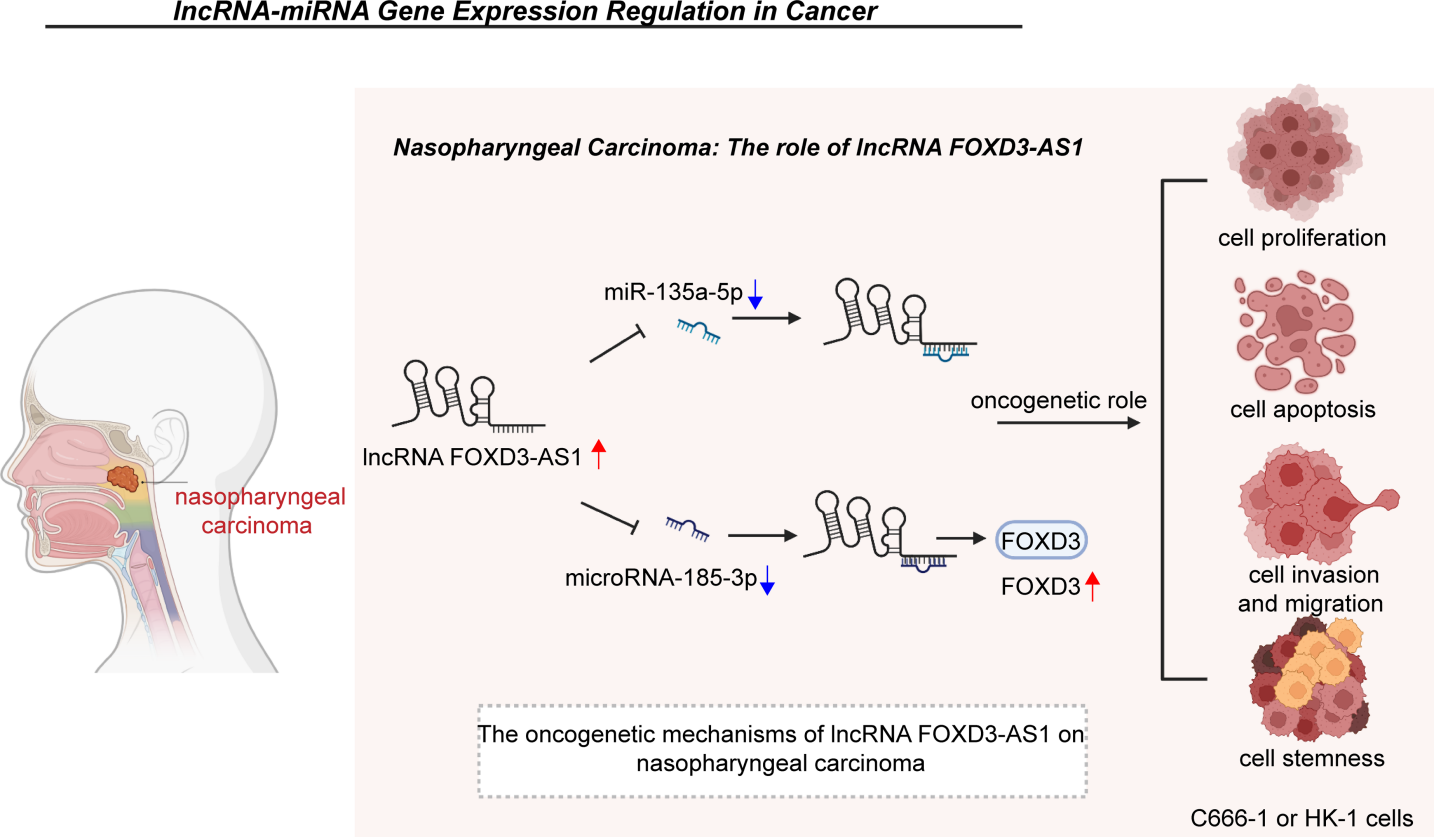
In nasopharyngeal carcinoma, FOXD3-AS1 promotes cell proliferation, apoptosis, invasion, migration and stemness by restraining the expression of miR-135a-5p or microRNA-185-3p and elevating the expression of FOXD3. Clearance of miR-30c modulates the expression of CTHRC1 and thus enhances the proliferative, invasive, and migratory abilities of MDA-MB-468 cells. LINC00707 also interacts with miR-206 to upregulate ER-α expression, which induces the proliferation and apoptosis of MCF-10AT cells.

Nasopharyngeal carcinoma is an endemic carcinoma in Southern China and is often diagnosed at an advanced stage (78–81). Effective markers for its early diagnosis are urgently needed to improve patient survival and reduce mortality rates (82–84). The expression level of FOXD3-AS1 was found to be upregulated in nasopharyngeal carcinoma tissues and cell lines (C666-1 and HK-1) and positively associated with tumor node metastasis (TNM) stage and a more invasive pathological classification. FOXD3-AS1 was shown to regulate cell proliferation, apoptosis, invasion, migration, cell stemness and tumor growth in a xenograft model in nude mice (33, 41) (**Figure 1**).

#### Colorectal Cancer

Colorectal cancer is the most common cancer in the world, characterized by elevated incidence and mortality rates (85–87). Colon adenocarcinoma (COAD) is the most prevalent pathological subtype of colon cancers, constituting 98% of all newly diagnosed cases of colon cancers (88–92). Previous evidence has indicated that FOXD3-AS1 is upregulated in COAD tissues as well as in HCT116 and SW1116 cells, while its expression correlates with key clinical features, including: TNM stage, poor tumor differentiation, lymph node metastasis, overall survival and progression-free survival (36). A series of functional experiments *in vitro* and *in vivo* have validated the oncogenic property of FOXD3-AS1 in colon cancer through of the regulation of cell proliferation, migration, invasion, and apoptosis. However, Tian Y et al. has also demonstrated that low expression of FOXD3-AS1 in colon cancer patients is associated with worse overall survival (93). Therefore, more research is needed to clarify the specific role of FOXD3-AS1 in colon cancer.

#### Melanoma

Melanoma is a highly aggressive and prevalent tumor, which exhibits a gradual upward trend in both morbidity and mortality rates (94–96). Thus, the identification of early diagnostic biomarkers and therapeutic targets remains urgent (97, 98). Increased expression of FOXD3-AS1 has been observed in melanoma tissues and cell lines (A2058, SK-MEL-28, SK-MEL-1, SK-MEL-2, and A375 cells) and positively associated with tumor size, AJCC stage, lymphatic metastasis and overall survival (37, 38). Functionally, high levels of FOXD3-AS1 facilitate cell proliferation, invasion and migration and repress cell apoptosis in A375, SK-MEL-1 and SK-MEL-2 cells. Moreover, experiments in murine models of xenograft tumor with accelerated tumor growth further validated the pro-oncogenic role of FOXD3-AS1 in melanoma.

#### Liver Cancer

Liver cancer is considered the sixth most frequent type of cancer, while hepatocellular carcinoma (HCC) is the most common type of liver cancer, consisting of 75–85% of all cases according to GLOBOCAN 2018 data (87, 99–101). Currently, serum AFP is the most widely used biomarker for HCC screening, despite its low sensitivity and a high false-negative rate for early HCC diagnosis. Therefore, it is crucial to explore novel biomarkers relevant for the early diagnosis and prognosis of HCC patients (102, 103). Indeed, the levels of FOXD3-AS1 have been shown to be significantly decreased in HCC tissues as well as in Huh7, Huh6, and SK-HEP-1 cells compared to health tissues or cells (39). FOXD3-AS1 expression has also been closely associated with poor prognosis of HCC patients. In addition, FOXD3-AS1 has been reported to significantly accelerate malignant processes of cell proliferation, invasion and migration in Huh6 cells, resulting in the development of HCC.

#### Osteosarcoma

Osteosarcoma is the most common primary bone tumor in children and young adults (104–108). However, the overall survival of patients with osteosarcoma remains unfavorable despite attempts to improve the efficacy of chemotherapy (109–111). Recent studies have proposed that targeting lncRNAs may provide a novel insight into the treatment of osteosarcoma (112–115). FOXD3-AS1 expression has been found to be upregulated in both osteosarcoma tissues and cell lines (U2OS, MG-63, HOS, SAOS2 and 143B cells) (35). *In vitro* studies revealed that knockdown of FOXD3-AS1 dramatically impaired the invasion and migration of osteosarcoma cells. *In vivo* nude mice models have further confirmed that FOXD3-AS1 favors the development and progression of osteosarcoma.

#### Thyroid Cancer

It was reported that FOXD3-AS1 was overexpressed in thyroid cancer tissues and FTC-133, SW579, TPC-1, 8505C cells (40). Furthermore, upregulated FOXD3-AS1 expression has been demonstrated to accelerate thyroid tumor growth in *in vivo* xenograft models and intensify the biological processes of cell proliferation, invasion and migration in FTC-133 cells.

#### Neuroblastoma

Neuroblastoma is the most common pediatric malignancy, accounting for 15% of tumor-related deaths in children (116–119). Although there have been some advancements in the management of neuroblastoma patients, the 5-year event-free survival rate for high-risk groups is still poor (120, 121). Indeed, FOXD3-AS1 was shown to be downregulated in neuroblastoma tissues and NB-1643, SK-N-BE (2), NB-1691, IMR32, and BE (2)-C cells, and was regarded as an independent biomarker for a favorable prognosis (41). Experiments *in vitro* indicated that FOXD3-AS1 strongly accelerated neuronal differentiation and impaired the proliferation and invasiveness of IMR32 and BE (2)-C cells. Additionally, FOXD3-AS1 expression was inversely correlated with the growth rate of neuroblastoma in an *in vivo* xenograft model. In this same model, FOXD3-AS1 expression was correlated with a longer survival time of nude mice, corroborating the tumor suppressor roles of FOXD3-AS1 in neuroblastoma.

#### Glioma

Previous evidence has shown that FOXD3-AS1 is upregulated in glioma tissues and U87, A172 and U251 cells. Moreover, a higher FOXD3-AS1 expression was observed in high-grade glioma tissues when compared to that of low-grade glioma tissues (42). FOXD3-AS1 expression has been positively correlated to poorer overall survival and worse tumor grade. More importantly, FOXD3-AS1 has been proposed as an oncogene, favoring the proliferation, invasion and migration of U251 and A172 cells.

### Non-Cancer Disease

#### Ischemic Stroke

Ischemic stroke accounts for one of the most impactful diseases worldwide, leading to high mortality and disability rates (122–125). Due to the narrow time window and ischemia-reperfusion (I/R) injury, the effect of vascular recanalization and reperfusion treatment has been limited (126–128). Therefore, exploring the exact molecular pathways underlying I/R injury is a pressing concern (129–132). Upon I/R injury, FOXD3-AS1 was found to be overexpressed *in vivo* and *in vitro* in oxygen-glucose deprivation/reoxygenation (OGD/R)-induced neuro-2A (N2a) cells (45). Furthermore, FOXD3-AS1 knockdown exerted neuroprotective effects in ischemic stroke by inhibiting neuronal cell apoptosis and cerebral infarction, in addition to facilitating neuronal functional recovery.

#### Myocardial Disease

Myocardial ischemia is the major cause of cardiovascular morbidity and mortality in the world (133–135). The pathophysiological process of myocardial I/R injury results in the deficiency of oxygen supply to myocardial cells and subsequent development of oxidative stress, which is vital for energy metabolism, cardiac dysfunction and cell death (136, 137). Therefore, the mitigation of myocardial I/R is needed to improve the quality of life and reduce the mortality of these patients (138, 139). High levels of FOXD3-AS1 were found in H9C2 cells subjected to OGD/R during myocardial I/R injury and in hypoxic AC16 cells (46, 47). More importantly, FOXD3-AS1 has been demonstrated to induce cardiomyocyte autophagy and aggravate the apoptosis of H9C2 cells, contributing to myocardial I/R injury. Additionally, FOXD3-AS1 knockdown was shown to protect AC16 cardiomyocytes against I/R injury by increasing cell survival and inhibiting apoptosis.

#### Acute Respiratory Distress Syndrome

**Figure 2:**
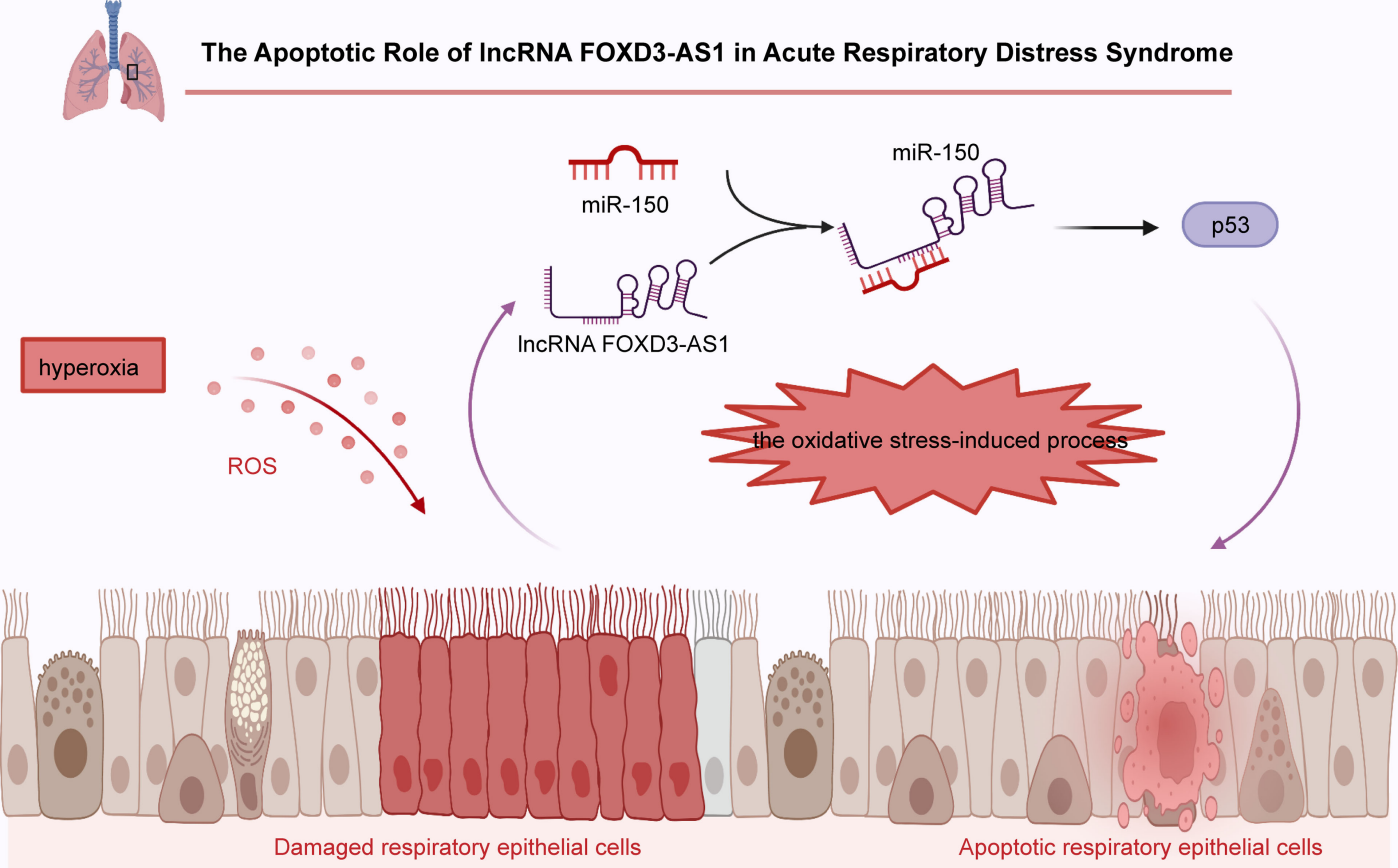
In acute respiratory distress syndrome, FOXD3-AS1 accelerates oxidative stress-induced cell apoptosis by suppressing miR-150 and increasing p53 expression.

Acute respiratory distress syndrome (ARDS) is a life-threatening clinical condition of acute respiratory failure (140–142). Early diagnosis and prompt initiation of treatment are associated with favorable clinical outcomes in ARDS patients (143–146). Considering that this is a highly heterogeneous syndrome, potential early biological makers are needed to improve the management of ARDS (146–149). Previous reports have shown that FOXD3-AS1 is strikingly overexpressed in lung tissues of an *in vivo* ARDS model (HALI models) as well as in alveolar epithelial cell line A549, lung bronchial epithelial cells Beas2B and mouse primary lung epithelial cells after exposure to hyperoxia (48). Similar to its role in myocardial hypoxic injury, FOXD3-AS1 was revealed to be involved in the development of oxidative stress upon lung injury by accelerating the apoptosis of A549 and Beas2B cells (**Figure 2**).

#### Allergic Rhinitis

Allergic rhinitis (AR) is a common disorder characterized by nasal itching, sneezing and congestion (150–152). A recent study reported that FOXD3-AS1 was severely downregulated in the nasal mucosa of AR patients and nasal epithelial cells (NECs) after LPS treatment (43). Additionally, FOXD3-AS1 was shown to participate in the development of AR and to protect patients from damage induced by Th2 immunoreaction in AR through the inhibition of IL-25 expression and secretion.

#### Retinal Infection With Toxoplasma Gondii-Ocular Toxoplasmosis

Toxoplasmosis is an infection caused by the Toxoplasma gondii parasite and currently available treatments for toxoplasmosis are inefficient (153). A better understanding of the pathogenesis of toxoplasmosis infection is required to develop novel drugs treatments (154, 155). FOXD3-AS1 was found to be downregulated in human retinal Müller cells during retinal infection with Toxoplasma gondii-ocular toxoplasmosis. This descriptive *in vitro* study suggests a role for FOXD3-AS1 in toxoplasmosis infection, however *in vivo* experiments are still needed (44).

## Relevant Mechanisms Regulated By Foxd3-AS1

FOXD3-AS1 has been proved to regulate a range of biological processes, including cell proliferation, apoptosis, invasion, migration, chemoresistance and endoderm differentiation. In the following section, we will recapitulate the main functions and corresponding mechanisms of FOXD3-AS1 in the development and progression of diseases.

### Cell Proliferation

Uncontrolled cell proliferation is the main characteristic of cancers (156–158). FOXD3-AS1 has been demonstrated to promote cell proliferation in NSCLC cells *via* sponging miR-135a-5p and further regulating CDK6 level (27, 143). FOXD3-AS1 was also shown to interact with miR-127-3p and upregulate the expression of MED28. The overexpression of ELAVL1 and activation of the PI3K/Akt pathway was also reported to be a possible pro-proliferative mechanism of FOXD3-AS1 in A549 cells (28). Contrarily, it was found that FOXD3-AS1 suppressed cell proliferation through the miR-150/SRCIN1 axis in A549 and H1229 cells (25). In breast cancer T47D and MCF-7 cells, FOXD3-AS1 cleared the expression of miR-363 and upregulated TFF1 expression and PI3K/Akt signaling, leading to cell proliferation (29). In cervical cancer, FOXD3-AS1 directly interacts with miR-296-5p and elevates HMGA1 levels mediated by transcription factor SP1, thereby enhancing the proliferative ability of HeLa and C33A cells (31, 32). In nasopharyngeal carcinoma C666-1 and HK-1 cells, FOXD3-AS1 acts as a promoter of cell proliferation *via* the inverse regulation of miR-135a-5p (33) or microRNA-185-3p and the upregulation of its downstream gene, FOXD3 (34). In colon adenocarcinoma, FOXD3-AS1 was shown to upregulate SIRT1 by clearing miR-135a-5p in HCT116 and SW1116 cells, which is suggestive of increased cell. In melanoma A375, SK-MEL-1 and SK-MEL-2 cells, FOXD3-AS1 facilitates cell proliferation through binding to miR-127-3p and upregulating the expression of FJX1 (37) or by an interaction with miR-325 (38), which then increases the expression of MAP3K2. In hepatocellular carcinoma, FOXD3-AS1 improves the expression of RICTOR and activates AKT signaling through an interaction with miR-335, thus exerting a pro-proliferative function in Huh6 cells (39). In thyroid cancer FTC-133 cells, it was confirmed that FOXD3-AS1 promotes cell proliferation by functioning as a miRNA sponge of miR-296-5p and therefore activating the TGF-β1/Smads signaling pathway (40). Finally, the specific regulatory mechanism of FOXD3-AS1 in glioma has not been thoroughly studied. It is thought that FOXD3-AS1 may enhance the proliferation of U251 and A172 cells by a partial regulation of FOXD3 expression (42).

### Cell Apoptosis

Apoptosis is a type of programed cell death that has been implicated in the development and occurrence of cancers (159–163). FOXD3-AS1 was demonstrated to suppress the apoptosis of NSCLC A549 and H1229 cells through functioning as a ceRNA for miR-135a-5p and elevating CDK6 expression (24). Similarly, in cervical cancer HeLa and C33A cells, FOXD3-AS1 acts as an oncogene and competitively binds to miR-296-5p, which dramatically increases the levels of HMGA1, thus restraining tumor cells apoptosis (32). FOXD3-AS1 was also confirmed to weaken cell apoptosis in nasopharyngeal carcinoma C666-1 and HK-1 cells by negatively regulating miR-135a-5p (33) or microRNA-185-3p and upregulating the level of FOXD3 (34). In colon adenocarcinoma, FOXD3-AS1 was found to protect HCT116 and SW1116 cells from apoptosis *via* the regulation of miR-135a-5p/SIRT1 axis (36). Moreover, it was found that FOXD3-AS1 enhanced cell apoptosis of melanoma A375, SK-MEL-1 and SK-MEL-2 cells through either the miR-325/MAP3K2 axis or the miR-127-3p/FJX1 axis (37, 38). More importantly, downregulation of FOXD3-AS1 was identified to suppress cell apoptosis and subsequent cerebral I/R injury in ischemic stroke N2a cells through inactivating the expression of miR-765 and facilitating BCL2L13 expression (45). A similar phenomenon has been observed in myocardial I/R injury H9C2 cells, in which FOXD3-AS1 promoted cell autophagy and further exacerbated cell apoptosis through the NF-κB/COX2/iNOS signaling pathway (46). Another reported pro-apoptosis mechanism of FOXD3-AS1 in I/R injury of AC16 cardiomyocytes occurs through the downregulation of miR-150-5p (47). In addition, FOXD3-AS1 negatively modulates miR-150 and upregulates its target p53 during oxidative stress in ARDS Beas2B and A549 cells (48).

### Cell Invasion and Migration

Metastasis is a complex process, in which malignant cells spread from the primary tumor to surrounding organs, forming secondary tumors. Full understanding of mechanisms that regulate metastasis is essential (164).

FOXD3-AS1 was shown to enhance the invasion and migration of NSCLC A549 and H1229 cells by interacting with miR-127-3p and increasing MED28 expression (27). However, in NSCLC A549 and H1229 cells, FOXD3-As1 repressed epithelial-mesenchymal transition (EMT) and invasion through the activation of ELAVL1-mediated PI3K/Akt pathway and the miR-150/SRCIN1 axis (28). FOXD3-AS1 was also found to attenuate the invasiveness of neuroblastoma IMR32 and BE (2)-C cells by inhibiting the expression of PARP1 and CTCF (41). In cervical cancer HeLa and C33A cells, FOXD3-AS1 accelerates invasion and migration by competitively binding to miR-128-3p and elevating LIMK1 expression (31) as well as through clearing miR-296-5p and subsequently increasing HMGA1 levels (32). It was also reported in osteosarcoma MG-63 and HOS cells that FOXD3-AS1 is able to promote migration and EMT through the activation of ELF1, which is mediated by an interaction with miR-296-5p and increased levels of ZCCHC3 (35). In addition, FOXD3-AS1 antagonizes the expression of miR-135a-5p and upregulates SIRT1 in colon adenocarcinoma HCT116 and SW1116 cells, thus contributing to cell invasion and migration (36). Similarly, in melanoma A375, SK-MEL-1 and SK-MEL-2 cells, FOXD3-AS1 facilitated cell migration *via* miR-127-3p/FJX1 and/or miR-325/MAP3K2 (37, 38). FOXD3-AS1 promotes the invasion and migration of hepatocellular carcinoma Huh6 cells by serving as a miR-335 sponge, enhancing RICTOR expression and activating the AKT signaling pathway (39). In thyroid cancer FTC-133 cells, FOXD3-AS1 positively regulated cell migration and invasion through the inhibition of miR-296-5p and upregulation of the TGF-β1/Smads signaling pathway (40). Moreover, it was demonstrated that FOXD3-AS1 also promotes invasion and migration of glioma U251 and A172 cells through a partial modulation of FOXD3 (42).

### Cell Chemoresistance

Insensitivity to chemotherapy is a primary cause of treatment failure and shortens the life expectancy of patients (165, 166). Therefore, there is an urgent need for thoroughly understanding its mechanisms in order to develop new strategies to prevent drug resistance (167, 168).

**Figure 3:**
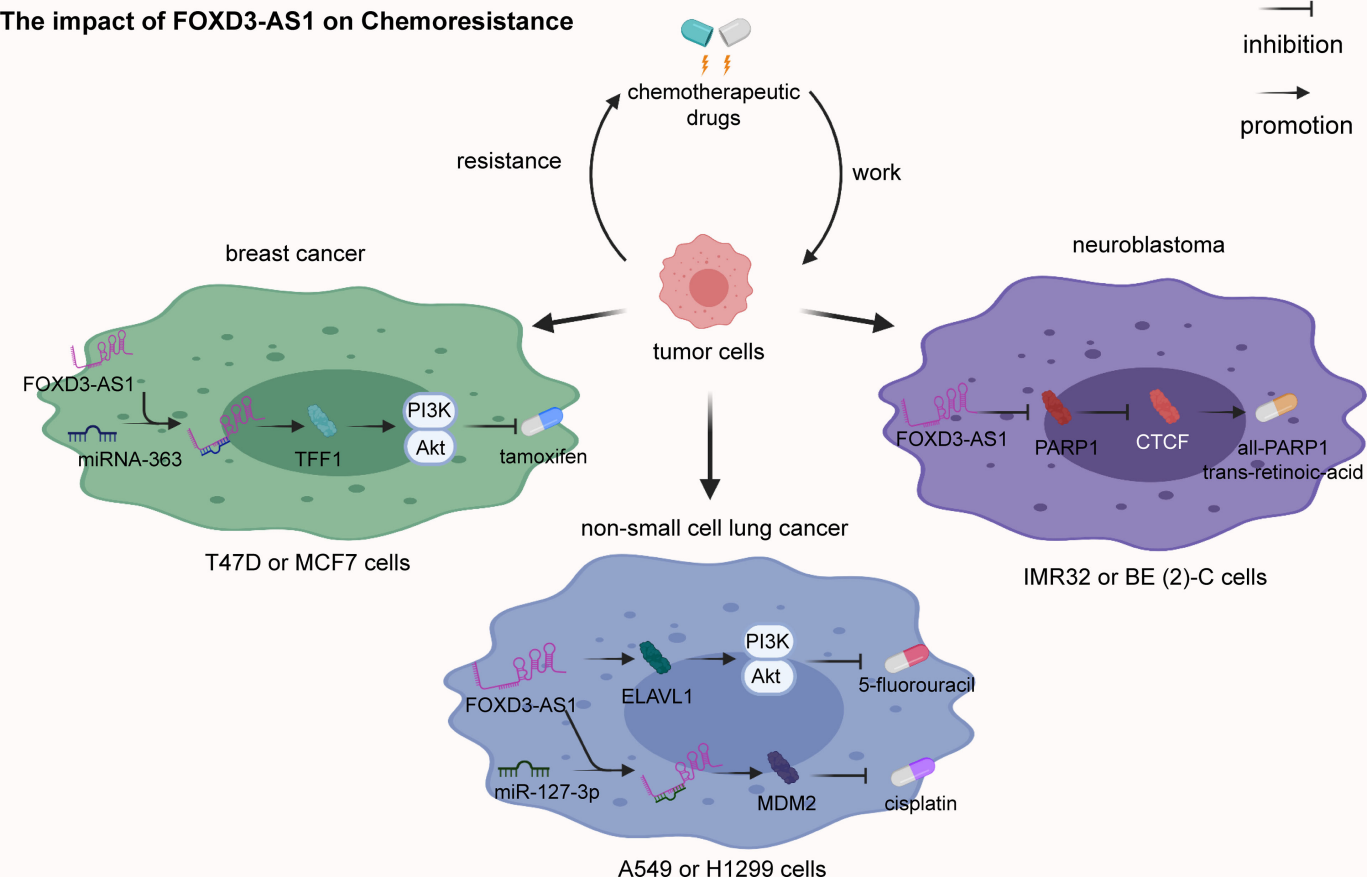
The impact of FOXD3-AS1 on chemoresistance. In non-small cell lung cancer A549 or H1299 cells, FOXD3-AS1 promotes cisplatin-resistance *via* inhibiting miR-127-3p expression and upregulating MDM2 expression. FOXD3-AS1 also enhances 5-fluorouracil resistance *via* activating ELAVL1 and the PI3K/Akt pathway. In breast cancer T47D or MCF7 cells, FOXD3-AS1 intensifies tamoxifen (TMX) resistance *via* clearing microRNA-363 and strengthening TFF1 and the PI3K/Akt signaling pathway. In neuroblastoma IMR32 or BE (2)-C cells, FOXD3-AS1 enhances the sensitivity of all-trans-retinoic-acid (ATRA) *via* repressing PARP1 and CTCF.

FOXD3-AS1 was found to promote cisplatin-resistance in NSCLC A549 and H1299 cells *via* the repression of miR-127-3p and subsequently increase of MDM2 (26). Moreover, FOXD3-AS1 has also been proved to intensify 5-fluorouracil resistance in NSCLC A549 cells through increasing ELAVL1 expression and the PI3K/Akt pathway (28). In addition, FOXD3-AS1 has been reported to enhance tamoxifen (TMX) resistance in breast cancer T47D and MCF7 cells through the microRNA-363/TFF1/PI3K/Akt signaling pathway (29). Notably, FOXD3-AS1 enhances the sensitivity of chemotherapeutic drugs in neuroblastoma IMR32 and BE (2)-C cells through repression of PARP1-mediated PARylation of CTCF (41) (**Figure 3**).

### Cell Stemness and Differentiation

Accumulating evidence suggests that cells with stem-like characteristics favor tumor development, such as metastasis and chemoresistance (169–172). Further investigation concerning the molecular biology of cancers is expected to promote the development of alternative therapies targeting the properties of cell stemness (173–176). FOXD3-AS1 was implicated in the regulation of stem-like properties of nasopharyngeal carcinoma C666-1 and HK-1 cells by inhibiting miR-185-3p expression and consequently increasing FOXD3 levels (34). FOXD3-AS1 also stimulates differentiation induced by all-trans-retinoic-acid (ATRA) on neuroblastoma IMR32 and BE (2)-C cells through the inhibition of PARP1 and CTCF (41). Additionally, FOXD3-AS1 was first thought to induce pluripotency and differentiation of human embryonic stem cell (hESCs), in which undifferentiated hESCs present a high expression of FOXD3-AS1, while endoderm and mesoderm differentiation is correlated with low FOXD3-AS1 expression (177). Downregulation of FOXD3-AS1 results in pluripotency dysregulation through the inhibition of endoderm pathways.

## Clinical Applications Of FOXD3-AS1

Based abovementioned mechanisms and effects of FOXD3-AS1, there has been increasing interest in using FOXD3-AS1 as a promising diagnostic or prognostic biomarker as well as therapeutic target for disease management.

### FOXD3-AS1 as a Diagnostic Biomarker

FOXD3-AS1 is abnormally expressed in multitude diseases, including NSCLC (24–28), breast cancer (29, 30), cervical cancer (31, 32), nasopharyngeal carcinoma (33, 34), osteosarcoma (35), colon adenocarcinoma (36), melanoma (37, 38), hepatocellular carcinoma (39), thyroid cancer (40), neuroblastoma (41), glioma (42), ischemic stroke (45), myocardial ischemia (46, 47), acute respiratory distress syndrome (48), allergic rhinitis (43) and retinal infection with toxoplasma gondii-ocular toxoplasmosis (44). Differential expression of FOXD3-AS1 on specific tissues is able to distinguish pathological tissues from adjacent normal ones, suggesting that FOXD3-AS1 can be a promising diagnostic marker for the early diagnosis of diseases. However, monitoring FOXD3-AS1 expression levels in tissues is an invasive and costly process for clinical practice. Detection of FOXD3-AS1 expression in body fluids, such as blood and urine will likely be more convenient for disease diagnosis.

### FOXD3-AS1 as a Prognostic Biomarker

Several studies have reported that FOXD3-AS1 is significantly associated with clinicopathological features, such as tumor size, tumor grade, TNM stage, poor differentiation of tumor tissues, lymph node metastasis, distant metastasis, survival probability, overall survival and progression-free survival. These characteristics indicate that FOXD3-AS1 can serve as a prognostic biomarker for clinical application. For example, high expression of FOXD3-AS1 has been proved to associate with poor International Federation of Gynecology and Obstetrics stage of cervical cancer, indicating that FOXD3-AS1 could be employed as an independent prognostic biomarker for the prediction of overall survival rates in cervical cancer (32). In particular, FOXD3-AS1 levels show a significant association with benign differentiation, International Neuroblastoma Staging System (INSS) stage, and MYCN amplification in neuroblastoma (41). Taken together, these features render FOXD3-AS1 the potential to be a reliable candidate for disease prognosis.

### FOXD3-AS1 as a Treatment Target

With recent advances in the understanding of FOXD3-AS1 in the pathogenesis of diseases, several relevant molecular mechanisms and signaling pathways may be suitable for targeted therapy. Substantial studies have shown that FOXD3-AS1 participates in cell proliferation, apoptosis, metastasis, cell stemness and chemoresistance of various human diseases. Especially, the effect of FOXD3-AS1 on drug resistance has received considerable attention.

Emerging studies have indicated that FOXD3-AS1 induces resistance to chemotherapy and subsequently accelerates the development of different types of cancer. For example, FOXD3-AS1 enhances the resistance of NSCLC cells to chemotherapeutic drugs cisplatin and 5-fluorouracil (26, 28). Moreover, it has also been demonstrated that FOXD3-AS1 was able to enhance breast cancer cells resistance to tamoxifen (29). However, FOXD3-AS1 has also been reported to enhance neuroblastoma cell sensitivity to ATRA (41). Strategies aimed at targeting FOXD3-AS1 and modulating drug resistance are expected to be a new breakthrough in drug development. However, significant challenges remain for the safety and efficacy of FOXD3-AS1-targeted agents due to the lack of sufficient clinical data. Therefore, more in-depth basic research into the function and mechanisms of FOXD3-AS1 in diseases is needed.

## Conclusion

Numerous studies have shown that FOXD3-AS1 is highly expressed in multiple diseases, including breast cancer, cervical cancer, nasopharyngeal carcinoma, osteosarcoma, colon adenocarcinoma, melanoma, hepatocellular carcinoma, thyroid cancer, glioma, ischemic stroke, congenital heart disease and acute respiratory distress syndrome. In addition, studies have also revealed that FOXD3-AS1 is downregulated in neuroblastoma and allergic rhinitis, suggesting a protective role. Of note, different reports of FOXD3-AS1 in NSCLC show conflicting results on expression, which might be attributed to various factors, such as tumor heterogeneity (178), different study designs and insufficient number of tumor samples. The in-depth understanding of the differential expression of FOXD3-AS1 between normal and pathological tissues and cell lines might be expected to enhance the development of novel strategies for disease diagnosis. Further research with additional cell lines and animal models are needed to fully explore these differences. Moreover, the expression levels of FOXD3-AS1 show a close association with clinicopathological features, such as tumor size, grade, poor differentiation, lymph node metastasis, distant metastasis, overall survival and progression-free survival, which might be available for predicting the prognosis of patients. Mechanistic studies have reported that FOXD3-AS1 promotes cell proliferation, apoptosis, invasion, migration, chemotherapeutic resistance, cell stemness and differentiation. Functional studies of FOXD3-AS1 in recent years have broadened our knowledge of its regulatory mechanisms in disease and brought new perspectives on the clinical applications of FOXD3-AS1. Compared with conventional chemotherapy, molecular-targeted FOXD3-AS1 therapy is expected to show greater specificity and lower systemic toxicity. However, these therapies are still in early stages. The lack of support from clinical trials and toxicological experiments remains a major challenge for the applications of FOXD3-AS1. In addition, the stability and levels of FOXD3-AS1 in serum or other accessible biological samples have yet to be validated. Further molecular mechanisms and larger clinical multicenter studies should be conducted.

## Author Contributions

DC provide a source of ideas for this review. XZ collected the related paper. QY drafting and reviewed the manuscript. All authors have contributed substantially to original research and approved the submitted version.

## Funding

This work was funded by the National Nature Science Foundation (81802085).

## Conflict of Interest

The authors declare that the research was conducted in the absence of any commercial or financial relationships that could be construed as a potential conflict of interest.

## Publisher’s Note

All claims expressed in this article are solely those of the authors and do not necessarily represent those of their affiliated organizations, or those of the publisher, the editors and the reviewers. Any product that may be evaluated in this article, or claim that may be made by its manufacturer, is not guaranteed or endorsed by the publisher.
